# Global and Complement Gene-Specific DNA Methylation in Grass Carp after Grass Carp Reovirus (GCRV) Infection

**DOI:** 10.3390/ijms19041110

**Published:** 2018-04-07

**Authors:** Lv Xiong, Libo He, Lifei Luo, Yongming Li, Lanjie Liao, Rong Huang, Zuoyan Zhu, Yaping Wang

**Affiliations:** 1 State Key Laboratory of Freshwater Ecology and Biotechnology, Institute of Hydrobiology, Chinese Academy of Sciences, Wuhan 430072, China; 18963973548@163.com (L.X.); luolifei145@sina.com (L.L.); liym@ihb.ac.cn (Y.L.); liaolj@ihb.ac.cn (L.L.); huangrong@ihb.ac.cn (R.H.); zyzhu@ihb.ac.cn (Z.Z.); 2 University of Chinese Academy of Sciences, Beijing 100049, China

**Keywords:** grass carp, grass carp reovirus, DNA methylation, gene expression, complement component 3, kininogen-1, negative correlation

## Abstract

Grass carp reovirus (GCRV) causes huge economic loss to the grass carp cultivation industry but the mechanism remains largely unknown. In this study, we investigated the global and complement gene-specific DNA methylation in grass carp after GCRV infection aimed to uncover the mechanism underlying GCRV infection. The global DNA methylation level was increased after GCRV infection. Expression levels of enzymes involved in DNA methylation including DNA methyltransferase (DNMT), ten-eleven translocation proteins (TETs), and glycine *N*-methyltransferase (GNMT) were significantly altered after GCRV infection. In order to investigate the relationship between the gene expression level and DNA methylation level, two representative complement genes, complement component 3 (*C3*) and kininogen-1 (*KNG1*), were selected for further analysis. mRNA expression levels of the two genes were significantly increased at 5 and 7 days after GCRV infection, whereas the DNA methylation level at the 5′ flanking regions of the two genes were down-regulated at the same time-points. Moreover, a negative correlation was detected between gene expression levels and DNA methylation levels of the two genes. Therefore, the current data revealed a global and complement gene-specific DNA methylation profile after GCRV infection. Our study would provide new insights into understanding the mechanism underlying GCRV infection.

## 1. Introduction

Grass carp (*Ctenopharyngodon idellus*) is an important aquaculture species in China, accounting for about 18% of total freshwater aquaculture production. The production of grass carp reached 5.5 million tons in 2014, making it the most highly consumed freshwater fish worldwide [1]. Unfortunately, frequent diseases always result in huge economic loss to the grass carp cultivation industry. Grass carp hemorrhagic disease which is caused by the grass carp reovirus (GCRV) is one of the most serious problems that hamper the development of grass carp aquaculture [2]. Therefore, GCRV has been noticed with special concern by fish breeders and immunologists with the hope of developing disease-resistant breeding or understanding the mechanism of GCRV infection [3].

DNA methylation refers to the addition of a methyl group to the 5’ position of a cytosine base to form 5-methylcytosine, and is one of the well characterized epigenetic markers in biota [4]. In general, DNA methylation in the cytosine-phosphor-guanine (CpG) sites of promoter regions or CpG islands of genes may be associated with gene expression levels [5,6]. Moreover, DNA hypermethylation in gene promoters is usually linked to transcriptional repression whereas hypomethylation is always correlated with transcriptional activation [7,8,9]. For example, the DNA methylation of the *cyp19a1a* gene in rare minnow under bisphenol A exposure was negatively correlated with gene expression levels [10]. mRNA expression levels of the *RIG-1* gene in grass carp after GCRV infection is negatively regulated by DNA methylation [11]. The CpA/CpG methylation of *CiMDA5* of grass carp negatively regulates its mRNA expression and is tightly associated with resistance against GCRV [12]. Low salinity affects the expression level of the *igf1* gene in the liver of half smooth tongue sole, whereas methylation level shows an opposite trend to that of gene expression levels [13].

DNA methyltransferase (DNMT) proteins catalyze the addition of a methyl group to the 5’ position of cytosine at a CpG site [14]. In vertebrates, three DNMTs including DNMT1, DNMT2, and DNMT3 have been described [15]. DNMT1 is involved in the maintenance of methylation profiles during DNA replication [16], DNMT3 is important in establishing new methylation patterns of DNA and is hence referred to as the de novo methyltransferase [17], while the role of DNMT2 is still unclear. Interestingly, six *DNMT3* family genes named *DNMT3* to *DNMT8* were reported in zebrafish, which is used as a model of teleost fish [18]. Beside the DNMTs, the ten-eleven translocation proteins (TETs) and glycine *N*-methyltransferase (GNMT) are two important enzymes that directly affect the DNA methylation level [19,20,21]. Up to now, three TET homologs (TET-1, TET-2, and TET-3) that share structural domains with their mammalian counterparts have been identified in zebrafish [22].

Due to the great economic loss that is caused by GCRV, many studies have been conducted about GCRV [23,24,25,26,27,28,29], but the mechanism underlying GCRV infection is still largely unknown. In the present study, we aimed to uncover the mechanism of GCRV infection from the point of view of epigenetics. Grass carp were infected with GCRV and the global DNA methylation level was measured. The expression levels of enzymes that are involved in DNA methylation were examined. Moreover, two representative complement genes, complement component 3 (*C3*) and kininogen-1 (*KNG1*), were selected for analysis of the relationship between gene expression level and DNA methylation level after GCRV infection. Our work provides valuable information for understanding the mechanism of GCRV infection.

## 2. Results

### 2.1. Grass Carp after GCRV Infection

**Figure 1:**
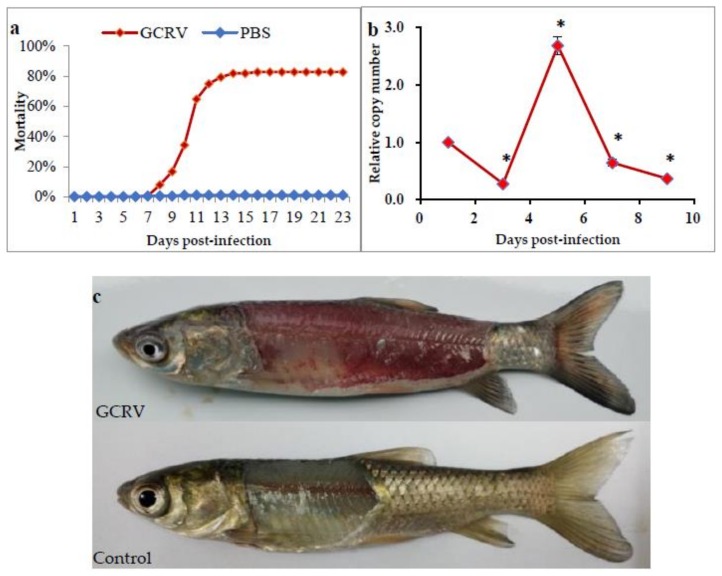
The fish after grass carp reovirus (GCRV) infection. (**a**) Cumulative mortality of fish. Fish were infected with 200 µL GCRV or PBS by intraperitoneal injection, respectively. The number of dead fish in each group was counted every day until no deaths were recorded for seven consecutive days. (**b**) Relative copy number of GCRV in infected fish. Data represent mean ± standard deviation of three replicates. Significant difference (*p* < 0.05) is indicated with asterisks (*). (**c**) The clinical symptom of fish after GCRV infection.

The grass carp were divided into two groups and intraperitoneally injected with GCRV or PBS, respectively. As shown in Figure 1a, for the group that was injected with GCRV, a total mortality of 82.8% was reached at 16 days, with the first death recorded as early as 8 days post-infection. In the group that was injected with PBS, three dead fish were observed, giving a total mortality of 1.3% (Figure 1a). To determine dynamic changes of GCRV in the infected group, the viral load in the GCRV-infected fish at different times after challenge was examined by qPCR. The relative copy number of GCRV at 1-day post-infection was used as a reference for normalization. As shown in Figure 1b, extremely low copy numbers of GCRV were observed at 3 days post-infection, whereas a marked increase was detected at 5 days post-infection. The viral copy number declined at 7 and 9 days post-infection. Moreover, typical hemorrhagic symptoms were observed in the GCRV-infected group, especially in the muscle and tail fin, while no hemorrhagic symptoms were present in the control group (Figure 1c). Thus, these results confirmed the efficiency of GCRV infection.

### 2.2. Global DNA Methylation Changes after GCRV Infection

**Figure 2:**
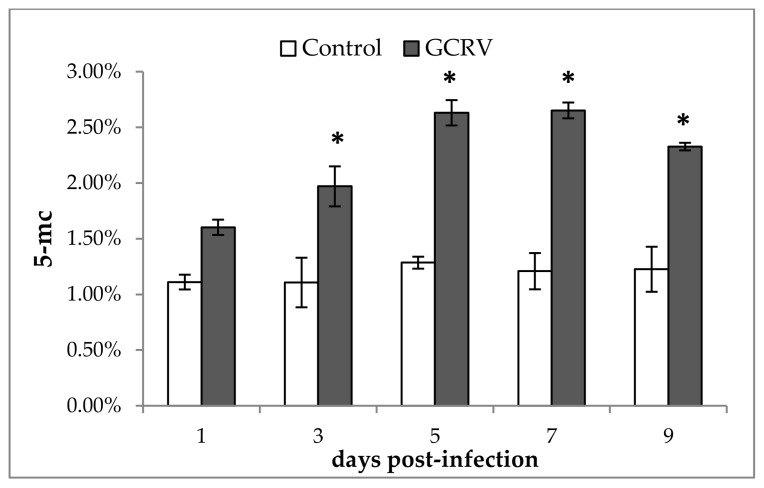
Global DNA methylation level of grass carp after GCRV infection. The spleen samples of two groups at different time points were obtained and global DNA methylation level was analyzed. Data were shown as the mean ± standard deviation of three replicates (*n* = 5 for each biological duplicate). Significant difference (*p* < 0.05) in DNA methylation level between the two groups are indicated with an asterisk (*).

In order to investigate whether the global DNA methylation was altered after GCRV infection, spleen samples of the two groups at different time points post-infection were obtained and global DNA methylation level was measured. As shown in Figure 2, for the control group that injected with PBS, the global DNA methylation level was almost consistent throughout the experiment. However, for the GCRV infected group, the percent of 5-methylcytosine (5-mc) was significant higher than that of control group at 3, 5, 7, and 9 days post-infection, while no significant difference was observed at the first day post-infection. Therefore, these results implied that the global DNA methylation level was altered after GCRV infection.

### 2.3. Expression Patterns of DNA Methyltransferase Genes after GCRV Infection

**Figure 3:**
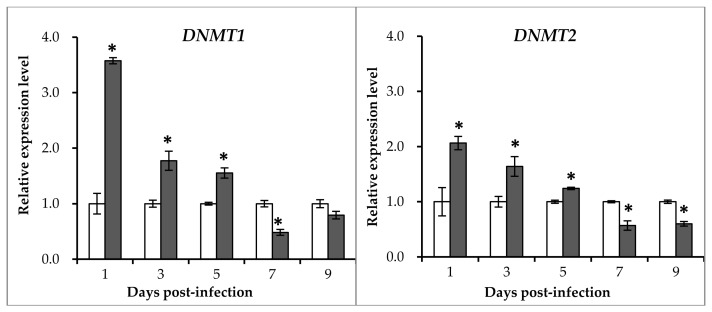

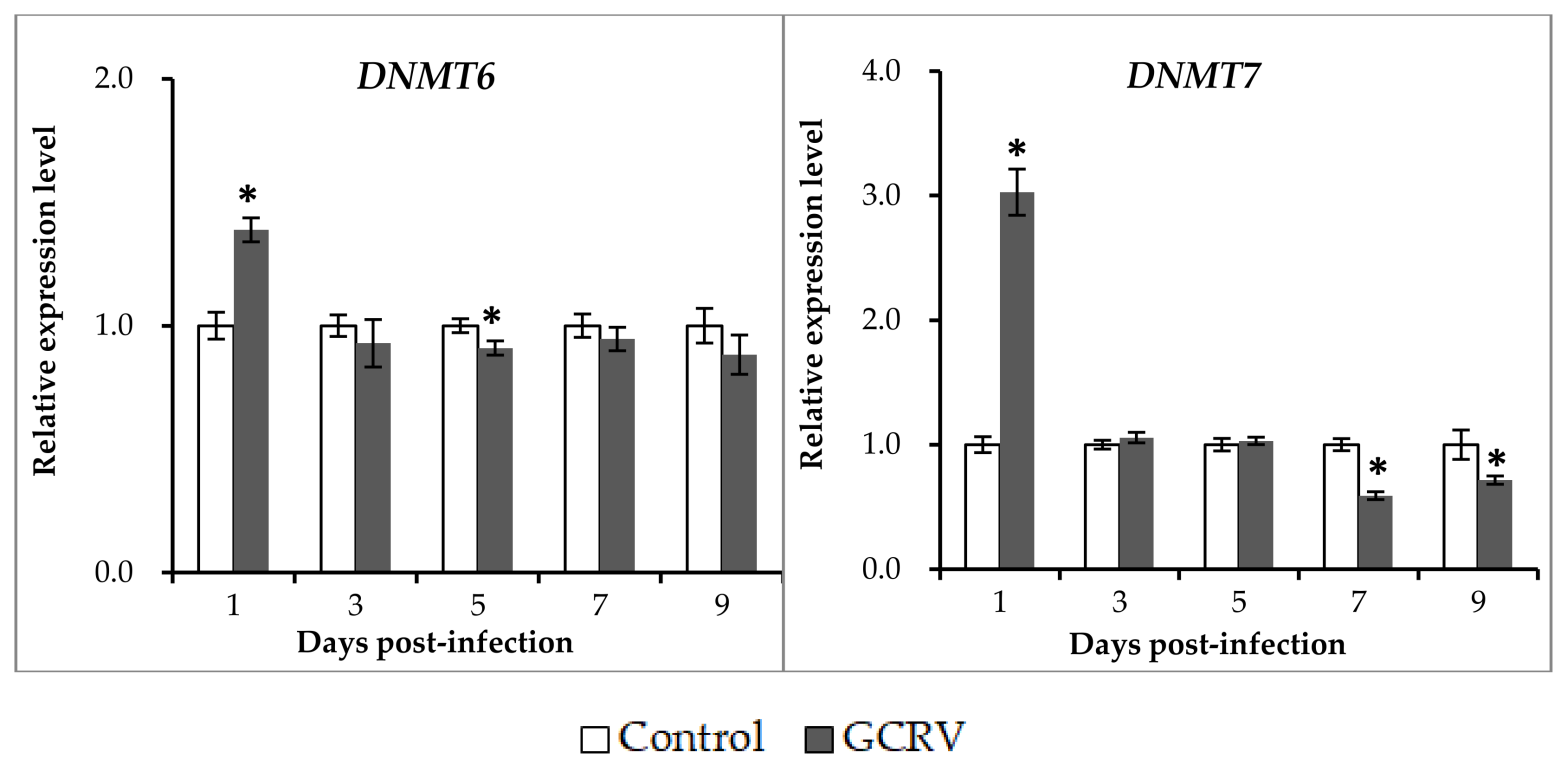
qRT-PCR analysis of the expression levels of DNA methyltransferases (*DNMT*s) after GCRV infection. The relative expression levels of genes were calculated as the ratio of gene expression levels in the GCRV-infected group relative to those in the control group at the same time points. All data represent the mean ± standard deviation of three replicates (*n* = 5 for each biological duplicate). Significant difference (*p* < 0.05) in gene expression levels between the two groups are indicated with an asterisk (*).

DNA methylation levels are associated with DNA methyltransferase (DNMT), which catalyzes the addition of a methyl group to the 5’ position of cytosine at a CpG site. Therefore, the expression patterns of *DNMT1*, *DNMT2*, and two *DNMT3* family genes (*DNMT6* and *DNMT7*) were examined after GCRV infection. As shown in Figure 3, overall, *DNMT1* and *DNMT2* genes showed similar expression patterns after exposure to GCRV while the expression patterns of *DNMT6* and *DNMT7* were distinct from them. To be specific, *DNMT1* and *DNMT2* showed a gradual decline from 1 day to 9 days post-infection, *DNMT6* and *DNMT7* presented a sharp peak at day 1 and reverted back to a basal level at 3 days post-infection. Hence, these results confirmed the alteration of *DNMT* gene expression levels after a GCRV challenge.

### 2.4. Expression Patterns of Ten-Eleven Translocation Proteins and Glycine N-methyltransferase after GCRV Exposure

**Figure 4:**
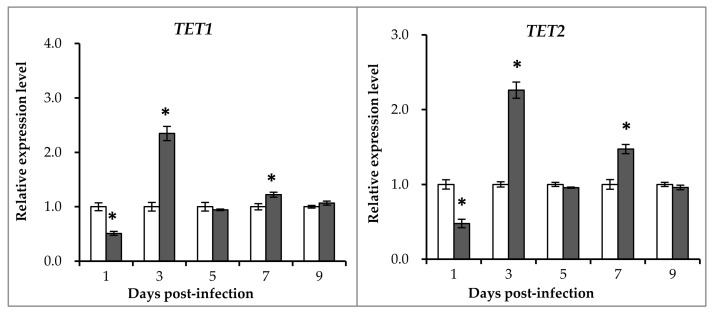

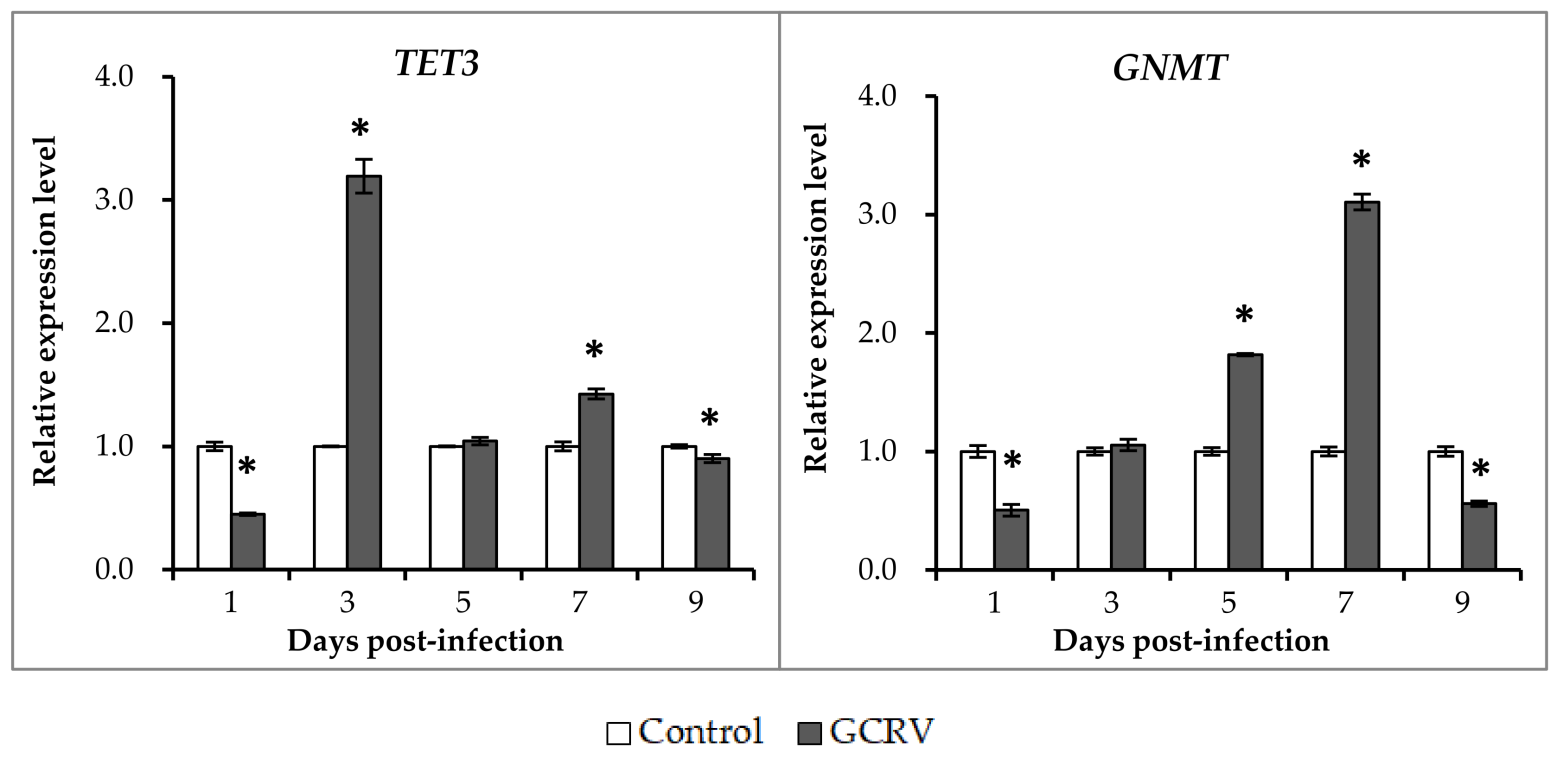
qRT-PCR analysis of the expression level of ten-eleven translocation proteins (*TET*s) and glycine N-methyltransferase (*GNMT)* after GCRV infection. The relative expression levels of genes were calculated as the ratio of gene expression levels in the GCRV-infected group relative to those in the control group at the same time-points. All data represent the mean ± standard deviation of three replicates (*n* = 5 for each biological duplicate). Significant differences (*p* < 0.05) in gene expression levels between the two groups are indicated with an asterisk (*).

In addition to *DNMT*s, ten-eleven translocation proteins (TETs) and glycine *N*-methyltransferase (GNMT) have also been reported to directly affect DNA methylation levels. Therefore, the expression patterns of three *TETs* (*TET-1*, *TET-2*, and *TET-3*) and the *GNMT* gene were investigated in this study. As shown in Figure 4, similar expression patterns were observed for the three *TET* genes. The expression levels of the three genes in the GCRV-infected group were lower than those of the control group at the first days post-infection, and higher than the control group at 3 and 7 days post-infection. For the *GNMT* genes, expression levels in the infected group were significantly higher than those in the control group at 5 and 7 days post-infection and slightly lower than the control group at the first and the last days (1 and 9 days post-infection).

### 2.5. Expression Patterns of Representative Complement Genes after GCRV Infection

**Figure 5:**
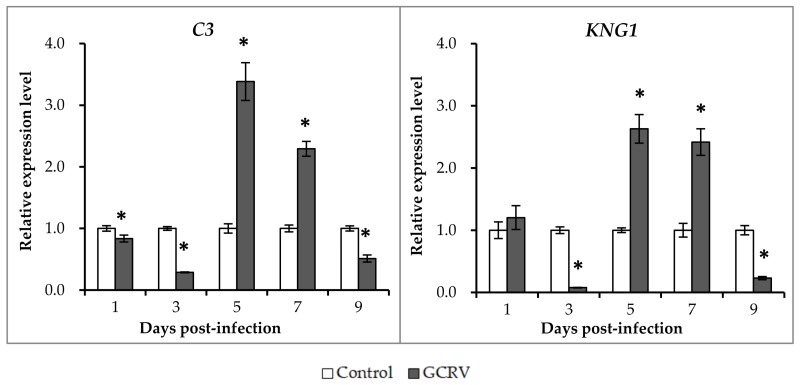
qRT-PCR analysis of the expression levels of *C3* and *KNG1* after GCRV infection. The relative expression levels of the genes were calculated as the ratio of gene expression level in GCRV infected group relative to that in the control group at the same time-point. All data represent the mean ± standard deviation of three replicates (*n* = 5 for each biological duplicate). Significant difference (*p* < 0.05) in gene expression level between the two groups are indicated with an asterisk (*).

In our previous study, we showed that the activation of the “complement and coagulation cascade” pathways may account for the hemorrhagic symptoms after GCRV infection [29]. In the present study, two representative complement genes, *C3* and *KNG1*, were selected to investigate their expression patterns after GCRV infection. As shown in Figure 5, a similar expression profile was observed for the two complement genes. The expression levels of the two genes were similar between the two groups at the first days after GCRV infection, whereas it was decreased in the GCRV-infected group at 3 days post-infection when compared to control group. However, a remarkable increase of the expression level was detected at 5 and 7 days post-infection in the infected group when compared with the control group. This increase was not persistent in the infected group, and declined at 9 days post-infection.

### 2.6. Methylation Changes in the 5′ flanking Region of C3 and KNG1 after GCRV Infection

**Figure 6:**
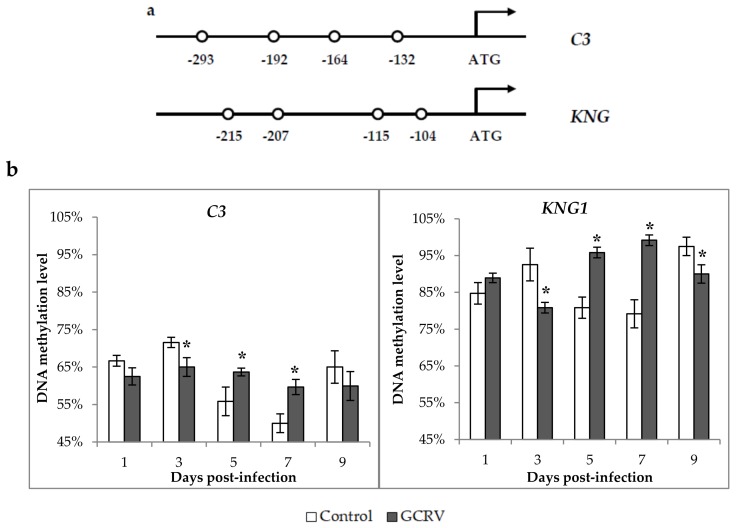
The DNA methylation level in the 5′ flanking region of *C3* and *KNG1* after GCRV infection. (**a**) A schematic diagram of CpG loci in the 5′ flanking region of *C3* and *KNG1* genes. Four CpG loci were selected in both *C3* (-293, -192, -164, and -132) and *KNG1* (-215, -207, -115, and -104) genes for BSP-PCR. (**b**) Changes of DNA methylation levels after GCRV infection. Data are shown as the mean ± standard deviation of three biological duplicates (*n =* 5 for each biological duplicate). Significant difference (*p <* 0.05) in DNA methylation level between the two groups are indicated with an asterisk (*).

The above results showed that the expression levels of two complement genes were significant altered after exposure to GCRV. To investigate whether the change of the expression levels were associated with the change of DNA methylation levels in the 5′ flanking regions of the two genes, bisulfite sequencing PCR was carried out for candidate CpG loci around the transcriptional start sites (TSSs) or translation initiation sites (TISs). As shown in Figure 6a, four representative CpG loci were selected in the 5′ flanking regions of both the *C3* and *KNG1* genes. The DNA of spleen samples from each group in each time-point were bisulfite treatment and subjected to bisulfite sequencing PCR. As shown in Figure 6b, for the *C3* gene, the DNA methylation level in the control group was slightly changed and ranged from 59.67 ± 2.02% to 65.00 ± 2.50% during the infection process. However, remarkable change of DNA methylation levels were observed in the GCRV-infected group. At 3 days post-infection, the DNA methylation level in the infected group was significantly higher than that of the control group (*p* < 0.05), while it was clearly decreased at 5 and 7 days post-infection (*p* < 0.05). For the *KNG1* gene, the methylation levels in both the infected and control groups were greater than that of *C3* gene. Specifically, DNA methylation levels showed no significant difference between the two groups during the first days post-infection. However, the methylation level in the infected group was significantly higher than that in the control group at 3 and 9 days post-infection (*p <* 0.05), whereas it declined at 5 and 7 days post-infection (*p <* 0.05).

### 2.7. The Relationship between Expression Level and DNA Methylation Level of C3 and KNG1 after GCRV Infection

**Figure 7:**
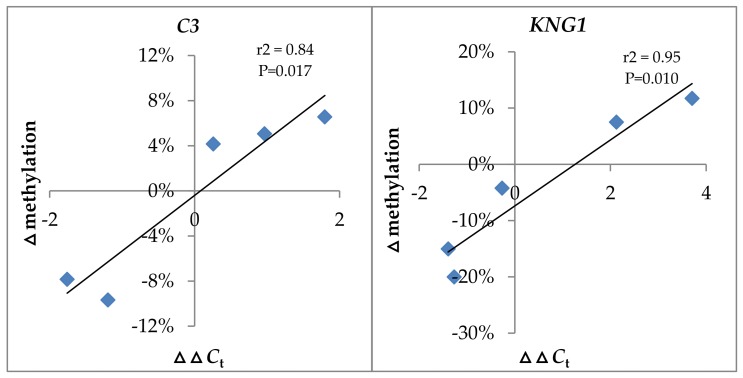
Correlation analysis between gene expression and DNA methylation levels of *C3* and *KNG1* after GCRV infection. The ΔΔ*C*_t_ value was used to evaluate gene expression levels whereas the Δmethylation between the infected and control groups was used to assess the DNA methylation level.

Based on the above results, we assumed a negative correlation between gene expression and DNA methylation levels for the two genes. To further confirm our hypothesis, correlation analysis was performed by the Statistical Product and Service Solutions (SPSS) software. The ΔΔ*C*_t_ value was used to evaluate the gene expression level whereas the Δmethylation between the infected and control groups was used to assess the DNA methylation level. As shown in Figure 7, for the *C3* gene, there is a significant positive correlation between ΔΔ*C*_t_ and Δmethylation (*r*^2^ = 0.84, *p* = 0.017). In addition, a similar result was also obtained for the *KNG1* gene (*r*^2^ = 0.95, *p* = 0.010). However, it should be mentioned that a high ΔΔ*C*_t_ value indicated a low gene expression level. Therefore, these results strongly suggest a negative correlation between gene expression and DNA methylation levels for the two genes.

## 3. Discussion

Epigenetics refers to heritable changes in gene function whereas DNA sequence does not change [30]. Epigenetic regulatory mechanisms include DNA methylation, histone modification, chromatin remodeling, and non-coding RNAs [31], of which DNA methylation is the most broadly studied and well-characterized epigenetic modification [32]. In the current study, the global and complement gene-specific DNA methylation in grass carp after GCRV infection were investigated in order to uncover the mechanism underlying GCRV infection.

Global DNA methylation measurement showed that the DNA methylation level in GCRV-infected group was significant higher than that of control group at 3, 5, 7, and 9 days post-infection, while no significant differences were observed in the first days post-infection. In general, DNA methylation in promoter regions was negatively correlated with gene expression level [5]. Therefore, the up-regulation of DNA methylation may suggest that the global gene expression level of the host was decreased after GCRV infection. After infection, normal host gene expression maybe hijacked or shutoff by the virus in order to benefit the virus’s own gene transcription and translation [33,34,35]. Therefore, it is clear that the global DNA methylation level was up-regulated after GCRV infection. A similar phenomenon was also reported in other viral infections. In the development of Epstein–Barr-virus-associated gastric carcinoma, DNA hypermethylation was observed [36]. The total DNA methylation level was significantly up-regulated in the thymus and bursa of chicken after H5N1 influenza viral infection (A-H5N1 strain) [37].

DNA methyltransferases (DNMTs) are important enzymes that are involved in DNA methylation [14]. Therefore, the expression profile of these genes was examined after GCRV infection. For the four *DNMT* genes that were examined, the expression levels in the infected group were significantly higher than those of the control group in the first days post-infection (Figure 3), whereas the global DNA methylation level showed no difference between the two groups at these time-points. In contrast to *DNMT*s, *TET*s are involved in demethylation which converts 5mC to 5-hydroxymethylcytosine (5hmC) and ultimately to a regular unmethylated cytosine residue [20,38]. GNMT catalyzes the *S*-adenosyl-l-methionine-(SAM)-dependent methylation of glycine to form sarcosine and plays important roles in the regulation of the cellular SAM/SAH ratio [39]. Therefore, the DNA methylation level may be the cooperation of these enzymes. Thus, it is not surprising that the global DNA methylation level was not in accordance with the expression level of *DNMT*s. In chickens before and after Marek’s disease virus (MDV) infection, the expression levels of *DNMT3a* and *DNMT3b* were also not consistent with the global DNA methylation level [40]. The global methylation level in the heart of young and middle-aged pigs showed significant differences, whereas no difference was detected in the expression levels of *DNMT1* and *DNMT3a* [41].

In our previous study, we showed that most of the genes involved in the “complement and coagulation cascades” pathways were significantly up-regulated at 7 days post GCRV infection, suggesting the activation of these pathways, which account for the hemorrhagic symptoms after GCRV infection [29]. Moreover, genes in these pathways were targeted by microRNAs [42], implying that epigenetic regulation took place after GCRV infection. To further reveal the epigenetic mechanisms, two representative complement genes were selected for bisulfite sequencing PCR to evaluate the methylation level in the infection process in spleen tissue. We chose spleen as the tissue for analysis because it is the major immune organ of fish and has a higher number of viral RNA copies [43]. Correlation analysis revealed that the DNA methylation level was negatively correlated with gene expression level. Therefore, these results suggested that complement genes after GCRV infection are not only targeted by microRNAs, but are also regulated by DNA methylation. Moreover, these results further prove that epigenetic regulation does indeed take place after GCRV infection. In addition, the viral copy number was determined in the infected fish (Figure 2b).It could be seen that a positive correlation between viral copy number and DNA methylation levels of both genes, suggesting the role of viral infection on the DNA methylation.

In conclusion, global and complement gene-specific DNA methylation in grass carp after GCRV infection was investigated. Global DNA methylation levels increased after GCRV infection. Expression levels of enzymes that are involved in DNA methylation were significantly altered after GCRV infection. Moreover, gene expression levels of representative complement genes were negatively correlated with DNA methylation levels after GCRV infection. Our work provides valuable information regarding the mechanism of GCRV infection.

## 4. Materials and Methods

### 4.1. Ethical Procedures

Animal welfare and experimental procedures were performed according to the Guide for the Care and Use of Laboratory Animals (Ministry of Science and Technology of China, 2006), and the protocol was approved by the committee of the Institute of Hydrobiology, Chinese Academy of Sciences (CAS). The reference number obtained was Y11201-1-301 (Approval date: 30 May 2016). All surgery was performed under eugenol anesthesia (Final concentration: 100 mg/L), and all efforts were made to minimize suffering.

### 4.2. Experimental Animals, Virus Exposure, and Sample Collection

Healthy grass carp, averaging 5 g in weight and 10 cm in length, were acclimatized in a circulating water system at 26–28 °C for 1 week and fed twice daily using a commercial diet. The photoperiod was 14 h:10 h light/dark and the dissolved oxygen in the water was maintained at 5–10 mg/L. Five grass carp were randomly selected for the PCR detection of GCRV with GCRV-specific primers (Table 1) to ensure that the fish were GCRV-free prior to the experiments. Then, the GCRV-free fish were divided into two groups (approximately 300 per group) that were maintained in separate tanks.

After no abnormal symptoms were observed in the two groups, viral challenge experiments were performed. In group I, fish were infected with GCRV (GCRV subtype II, 2.97 × 10^3^ RNA copy/µL) at a dose of 200 µL by intraperitoneal injection. In group II, fish were injected with an equal dose of PBS as a control group; 15 fish that contained three biological duplicates (*n =* 5 for each biological duplicate) from each group were collected and the spleens were removed for analysis at 1, 3, 5, 7, and 9 days post injection, respectively. For each biological duplicate, one half of the spleens were used to test the methylation level, while the other half was used to determine the gene expression level. Moreover, in order to calculate the total mortality in the two groups, the remaining fish in each group were monitored carefully and the number of dead fish was recorded. After no dead fish were recorded for seven consecutive days, the experiment was concluded and the total mortality was calculated.

### 4.3. DNA Isolation and Global DNA Methylation Measurement

Genomic DNA from the spleens of two groups was extracted using the traditional phenol–chloroform protocol with RNase treatment. DNA purity was checked using a NanoPhotometer spectrophotometer (IMPLEN, Munich, Germany) and the concentration was measured using the Qubit DNA Assay Kit with a Qubit 2.0 Flurometer (Life Technologies, Carlsbad, CA, USA). Global DNA methylation level was measured with the MethylFlash™ Global DNA Methylation (5-methylcytosine, 5-mC) ELISA Easy Kit (Epigentek, Brooklyn, NY, USA) according to the manufacturer’s protocol. The amount of input DNA for each assay was 100 ng to ensure optimal quantification. The analysis was performed on spleens of 15 grass carp (three biological duplicates, *n =* 5 for each biological duplicate) from each group in each time-point. For each biological duplicate, the DNA samples form five grass carp were mixed equivalently as a mixture for analysis. Data were shown as the mean ± standard deviation of three replicates.

### 4.4. Bisulfite Treatment and Sequencing

Bisulfite treatment of the DNA samples was carried out by using the EZ DNA Methylation-Gold™ Kit (Zymo Research, Irvine, CA, USA) according to the manufacturer’s instructions. In each biological duplicate, the DNA of five grass carp was mixed equivalently as a mixture for bisulfite treatment. Bisulfite-treated DNA was used for further analysis.

To verify whether the expression levels of complement genes were under methylation regulation, two representative complement genes, complement component 3 (*C3*) and kininogen-1 (*KNG1*), were selected. Genomic sequences of the two genes were extracted from the grass carp draft genome [44] and submitted to the online software Methprimer (http://www.urogene.org/cgi-bin/methprimer/methprimer.cgi) [45] to obtain the CpG island region and candidate CpG loci around the transcriptional start site (TSS) or translation initiation site (TIS). Specific methylation primers for bisulfite sequencing PCR were designed by Methprimer and verified again in Premier 5 software (Table 1). Moreover, an outer reverse primer was designed for each gene in order to improve the specificity of semi-nested BSP amplification. The DNA fragments were cloned into PMD18-T vector (TaKaRa, Shiga, Japan). Ten clones for each sample (biological duplicates) were randomly selected to sequence in order to evaluate the methylation status.

### 4.5. RNA Isolation and Quantitative Real-Time PCR (RT-qPCR)

TRizol reagent (Invitrogen, Carlsbad, CA, USA) was used to isolate RNA from spleen samples according the manufacturer’s protocol. The Qubit RNA assay kit (Life Technologies, Carlsbad, CA, USA) and RNA Nano 6000 assay kit (Agilent Technologies, Santa Clara, CA, USA) were used to measure the concentration and integrity of isolated RNA, respectively. DNase I (Promega, Madison, WI, USA)-treated total RNA was used as the template for the first-strand cDNA synthesis.

To investigate the gene expression levels of *DNMT1*, *DNMT2*, *DNMT6*, *DNMT7*, *TET-1*, *TET-2*, *TET-3*, *GNMT*, *C3*, and *KNG1*, RT-qPCR was carried out using a fluorescence quantitative PCR instrument (Bio-Rad, Hercules, CA, USA). The primer sequences are shown in Table 1. Each RT-qPCR mixture contained 10 μL 2× SYBR green master mix (Toyobo, Osaka, Japan), 7.4 μL ddH_2_O, 0.8 μL forward and reverse primers (for each primer), and 1 μL template. For each tested sample, three replicates (*n =* 5 for each biological replicates) were included. The *β-actin* gene was used as an internal control for normalization of gene expression. The program for RT-qPCR was as follows: 95 °C for 10 s, 40 cycles of 95 °C for 15 s, 58 °C for 15 s, and 72 °C for 30 s. The 2^−^^ΔΔ^*^C^*^t^ method was used to calculate relative expression levels [46]. Data represent the mean ± standard deviation (S.D.) of three replicates.

In addition, to determine the viral loads in the GCRV-infected group at different times after challenge, the relative copy numbers of the virus were examined by RT-qPCR using specific primers for the S6 segments of the GCRV (Table 1). The program and reaction mixture for qPCR were the same as above. Data were also represented as mean ± SD of three replicates.

### 4.6. Statistical Analysis

One-way ANOVA and Fisher’s least significant difference (LSD) posttest were used to determine the statistical significance between experimental groups and controls. Differences were considered significant at *p <* 0.05. Moreover, the correlation between ΔΔ*C*_t_ value of the two genes and methylation level of the CpGs in the 5′ flanking region of the two genes were examined using Pearson’s correlation coefficient in the Statistical Product and Service Solutions (SPSS) software; *p <* 0.05 was also considered for significant differences.

## Figures and Tables

**Table 1:** Primers used in the study.

Primers	Sequences (5′ to 3′)	Usage
GCRV-F	GGCGGCTGCTATGATACCAGTAGAC	GCRV detection
GCRV-R	GTCGCTTCAGATCAATCACCAAATC	
C3-BSP-F	TTAGAGGGTTATTATGTTGAGTATTT	BSP PCR of C3
C3-BSP-R	AAAACTCATAAAACCATTCAACTC	
C3-BSP-outer-R	AACAATATAAATTTTTCAAAAAATTC	
KNG1-BSP-F	TGTAAAGGTTTTTGTGAAGGTTTTT	BSP PCR of KNG1
KNG1-BSP-R	CCACTAAACTCTATCCTCCCCTATAATA	
KNG1-BSP-outer-R	TCTCTACTCTTCTAAAAACCCAACAA	
Qdnmt1-F	CCACCGAAATGTGCCGACTG	QPCR of dnmt1
Qdnmt1-R	GGTGCCCATGCTTGTCATACAC	
Qdnmt2-F	AAGTCTCAAAGCCCTTCACATTCTC	QPCR of dnmt2
Qdnmt2-R	ACTACCCCACTCCTGCCAAAAAC	
Qdnmt6-F	CAACCGCCACAGCCTCAAACT	QPCR of dnmt6
Qdnmt6-R	CCTCGGCCATCTCGTACTCTGAC	
Qdnmt7-F	GAGAAGCAAGTGGGCTCCTACG	QPCR of dnmt7
Qdnmt7-R	CAGCGATGGAGGTCGTAAGAGTT	
Qtet1-F	AGGCGTGTGAACATCAGGTGG	QPCR of tet1
Qtet1-R	AACCGAAAAACGGGGCATC	
Qtet2-F	CCTAACTCCAAAATAGACGGCAC	QPCR of tet2
Qtet2-R	GATGCGGGGTCATTGGTTTA	
Qtet3-F	GCCGCATATCCCTGGTCTTCTA	QPCR of tet3
Qtet3-R	TCGTGTCATCACACCGTCTCGT	
Qgnmt-F	AGTCTGAATGCCCTCTGGTGG	QPCR of gnmt
Qgnmt-R	ACCAGACTTCAAAGGGACCAAA	
QC3-F	ATGGTTCGCAAACACTCCTCAG	QPCR of C3
QC3-R	ACAGGTACTTGGCTTCTATGTCAACT	
Qkng1-F	TGAAGGTGCTCAACTGGCTCT	QPCR of kng1
Qkng1-R	AGTAACTCTTGACCTGCTTCTGTAAAC	
Q-β-actin-F	AGCCATCCTTCTTGGGTATG	QPCR of β-actin
Q-β-actin-R	GGTGGGGCGATGATCTTGAT	
Q-GCRV-F	AGCGCAGCAGGCAATTACTATCT	QPCR of GCRV
Q-GCRV-R	ATCTGCTGGTAATGCGGAACG	

## Abbreviations

C3	complement component 3
CpG	cytosine-phosphor-guanine
DNMT	DNA methyltransferase
GCRV	grass carp reovirus
GNMT	glycine *N*-methyltransferase
KNG1	kininogen-1
LSD	least significant difference
MDV	Marek’s disease virus
TETs	ten-eleven translocation proteins
TIS	translation initiation site
TSS	transcriptional start site
SAM	*S*-adenosyl-l-methionine
SPSS	Statistical Product and Service Solutions
5hmC	5-hydroxymethylcytosine
5-mC	5-methylcytosine
